# Dual adversarial deconfounding autoencoder for joint batch-effects removal from multi-center and multi-scanner radiomics data

**DOI:** 10.1038/s41598-023-45983-7

**Published:** 2023-11-01

**Authors:** Lara Cavinato, Michela Carlotta Massi, Martina Sollini, Margarita Kirienko, Francesca Ieva

**Affiliations:** 1 MOX, Department of Mathematics, Politecnico di Milano, Piazza Leonardo da Vinci, 32, Milan, 20133 Italy; 2 Health Data Science Centre, Human Technopole, Viale Rita Levi-Montalcini, 1, Milan, 20157 Italy; 3 Department of Biomedical Sciences, Humanitas University, Via Rita Levi Montalcini, 4, Pieve Emanuele, 20090 Italy; 4 Department of Nuclear Medicine, IRCCS Humanitas Research Hospital, Via Alessandro Manzoni, 56, Rozzano, 20089 Italy; 5 Fondazione IRCCS Istituto Nazionale dei Tumori, Via Giacomo Venezian, 1, Milan, 20133 Italy

**Keywords:** Cancer imaging, Data processing, Image processing, Machine learning, Statistical methods

## Abstract

Medical imaging represents the primary tool for investigating and monitoring several diseases, including cancer. The advances in quantitative image analysis have developed towards the extraction of biomarkers able to support clinical decisions. To produce robust results, multi-center studies are often set up. However, the imaging information must be denoised from confounding factors—known as batch-effect—like scanner-specific and center-specific influences. Moreover, in non-solid cancers, like lymphomas, effective biomarkers require an imaging-based representation of the disease that accounts for its multi-site spreading over the patient’s body. In this work, we address the dual-factor deconfusion problem and we propose a deconfusion algorithm to harmonize the imaging information of patients affected by Hodgkin Lymphoma in a multi-center setting. We show that the proposed model successfully denoises data from domain-specific variability (p-value < 0.001) while it coherently preserves the spatial relationship between imaging descriptions of peer lesions (p-value = 0), which is a strong prognostic biomarker for tumor heterogeneity assessment. This harmonization step allows to significantly improve the performance in prognostic models with respect to state-of-the-art methods, enabling building exhaustive patient representations and delivering more accurate analyses (p-values < 0.001 in training, p-values < 0.05 in testing). This work lays the groundwork for performing large-scale and reproducible analyses on multi-center data that are urgently needed to convey the translation of imaging-based biomarkers into the clinical practice as effective prognostic tools. The code is available on GitHub at this https://github.com/LaraCavinato/Dual-ADAE.

## Introduction

Hodgkin Lymphoma (HL) is a type of cancer that affects the lymphatic system, where lymphocytes proliferate uncontrollably in multiple lymph nodes and eventually in extranodal sites (e.g. spleen, bone, etc.). It is acknowledged as a curable disease thanks to its high rate of response to chemotherapy, often combined with radiotherapy. Still, a considerable percentage of patients do not respond to first-line treatments and the latest research has been devoting its efforts to discovering alternative and more efficient therapies, such as immunotherapy. Immunotherapy has indeed been approved for relapsing cases and has since represented a huge stride for patients, who are on average very young^1^.

As the number of available therapies increases, treatment planning becomes more and more crucial, and personalized medicine is catching on in every aspect of medical practice to devise the optimal treatment for each patient. Nevertheless, such a tailored approach requires quantitative and informative data to input into powerful and transferrable models on which to rely decisions. On purpose, Positron Emission Tomography/Computed Tomography (PET/CT) radiomic analysis has been shown to be an insightful, non-invasive tool for histological prediction, prognostic assessment, and bone marrow involvement definition in lymphoma^2^. In brief, the radiomics framework entails the extraction of a high-dimensional vector description of the spatial gray levels’ distribution of an image, the so-called radiomic features^3,4^. Each of such features thus describes a statistical property of the image heterogeneity at different scales, which can inform several downstream analyses and modeling efforts.

As HL is a rare disease, studies performed at a single institution usually do not account for sufficient information to build powerful enough models and derive general knowledge. Therefore, oftentimes multi-center cohorts need to be set up and large-scale studies have to be conducted, collecting data coming from different sources^5^. This raises a relevant issue, as radiomics features are known to be highly influenced by the image acquisition settings, the segmentation procedures, and the reconstruction parameters, jeopardizing the transferability and scalability of the results^6–8^. Typical exogenous confounding factors include both scanner characteristics, protocols and more general center-specific variabilities. These two factors must therefore be accounted for together when performing any type of analysis on multi-center data.

Moreover, the latest trend in radiomics is developing towards the extraction of more and more features, including first-order statistics, second- and higher-order statistics, and wavelet/frequency-derived indices. As the number of features rises, their pairwise correlation increases accordingly, and it becomes harder and harder to build effective models and disentangle the true signals of interest from technical artifacts, noise, and uninteresting biological variables. Here comes the need to properly reduce the dimensionality of radiomics vectors, transforming the features into low-dimensional vectors that keep the true informative signals while discarding domain-specific confounders.

While the above holds for many multi-center radiomics studies of (rare) diseases, when analyzing a hematological (like HL) or metastatic cancer, an additional level of complexity is added to the task of deconfounding and reducing radiomics features. In fact, different lesions can be found throughout the body of the patients. Despite the current approach for imaging-based quantitative assessment of most cancers, including HL, relies on the inspection of the bigger or hotter lesion, Sollini et al.^9^ have demonstrated how lesions are radiologically heterogeneous within patients in terms of radiomics description and how a prognostic classifier performs better when all tracer-avid lesions are considered. These findings align with the latest discoveries in the biological underpinnings of lymphomas. Some studies on solid cancers have previously described how both proximal and distant lesions deriving from the same primary tumors exhibit divergent patterns of both morphological and genetic heterogeneity^10^. Similarly, Tabanelli et al.^11^ reported the same evolutionary crossroad between morphological heterogeneity and intra-clonal evolution in a case of high-grade B-cell lymphoma. Thus, morphological heterogeneity behaves as a surrogate of genetic heterogeneity, responsible for treatment inefficacies. It follows that all lesions’ morphology must be taken into account, to exhaustively represent the disease in the prediction of cancer progression, therapy efficacy, and disease-free survival outcomes^12,13^. This implies that any postprocessing (i.e. dimensionality reduction and/or deconfusion process) aimed at preparing radiomics features for patients’ representations needs to keep the inter-lesion relationships within patients consistent, as here is where information of tumor morphological heterogeneity lies^9,14^.

In light of the above, a robust post-image-acquisition method aimed to harmonize multi-lesion radiomics data from multi-center studies requires (i) to properly remove both scanner and center confounding effects, (ii) to treat features’ collinearity and allow for simpler statistical modeling via proper dimensionality reduction, and (iii) to keep intra-patient heterogeneity consistent throughout the transformation. All this should be achieved while retaining all truly informative signals in the data, so as not to affect—and possibly improve—any potential downstream analysis.

Different strategies have been proposed in recent literature to minimize the batch-effects of radiomics variability, ranging from imaging-based to feature-based approaches^15–17^. Most of them aim to perform batch-specific standardization of images to disentangle the true signal from environment-related noise. Among these, the ComBat method was shown to be superior to other techniques, attracting attention in the radiomics field^15,18,19^. Starting from its first conception, ComBat was improved over time by different independent researchers. One for all, Adamer et al. proposed a regularized solution of ComBat, namely ReComBat, computationally more efficient to facilitate the large-scale harmonization of data^20^. However, it must be noted that ComBat and most of its derivative algorithms were developed in the computational biology domain, where usually only one main confounder (i.e. sequencing batch effect) needs to be removed. Indeed, to remove multiple confounders, they must be applied repeatedly, one factor at a time. As the context of radiomics studies oftentimes implies multiple confounders, Nested ComBat^21^ and its improved evolution from the same authors, OPNested Combat^22^, were recently proposed specifically to tackle multi-factor deconfusion. The latter applies ComBat iteratively on confounder-associated subsets of features, identifying the optimal order of factors to correct for. Notably, irrespective of the number of confounders removed from the data, ComBat-based methods rely on the hypothesis of normality of the features’ errors, which might be unrealistic for radiomics data^22^. Moreover, none of the above methods perform dimensionality reduction and are thus typically followed by Principal Component Analysis (PCA) before the analysis. Additionally, to the best of our knowledge, none of them has neither explicitly addressed the problem of preserving inter-lesion relationships within patients, nor has been evaluated in their capability to improve prediction by exploiting heterogeneity information after deconfusion.

**Figure 1:**
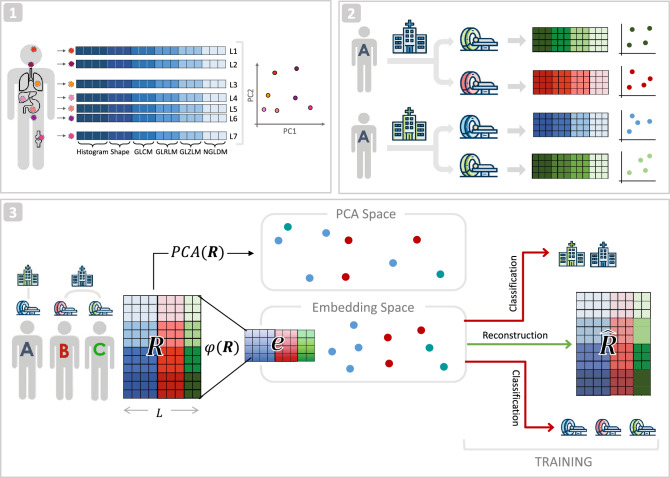
Graphical schema of multi-lesion multi-center radiomics studies confounding issues and Dual AD-AE solution: (**1**) In multi-lesion and/or metastatic tumor settings such as HL, patients can be modelled as clouds of points, where each point (*L*) is defined by the radiomic vector of a lesion (radiomic features can be semantically divided into histogram-derived, shape-derived, GLCM-derived, GLRLM-derived, GLZLM-derived and NGLDM-derived features). Among others, cloud heterogeneity is a valuable predictor of patients’ outcome. (**2**) The radiomics features computed for the same patient (Patient A) can be highly affected by the center that collects the images and the scanner used, which are the two primary confounding factors. In turn, this affects the patient’s cloud representation, biasing heterogeneity-based prediction and making patients with different center-scanner combinations hardly comparable. (**3**) To harmonize data from multi-center studies (matrix *R* of confounded radiomics features), Dual AD-AE embeds lesion into a lower dimensional space (Embedding Space) where exogenous confounding factors are removed and patients’ clouds keep the predictive information—as opposed to directly performing PCA on the combined dataset, which results in biased patients’ representations (Panel 3 top). To do that, the encoder of the Dual AD-AE ($\phi$) is trained to transform the radiomics matrix *R* into the embedding matrix *e* by maximizing the reconstruction (from embeddings to the reconstructed version of the input $\hat{R}$) and simultaneously minimizing the prediction on both center and scanner confounding factors (Panel **3** right).

In this work, we propose a multi-factor deconfusion algorithm better suitable for the downstream analysis of multi-lesion/metastatic patients in multi-center studies, described in Fig. 1. The algorithm builds upon the work of Dincer et al.^23^, which, in the context of gene expression analysis, proposes an adversarial deconfounding autoencoder (AD-AE) model that requires no assumption on features’ distribution and jointly performs dimensionality reduction and cleaning of the embeddings, enhancing the signal-to-noise ratio. Here, we exploit the rationale of this model for the context of multi-center PET/CT radiomics analysis, developing a Dual factor AD-AE (in the following, Dual AD-AE) model for the simultaneous removal of both center and scanner confounding effects (Fig. 1). We evaluate the proposed model in terms of (1) its deconfusion power, (2) its ability to keep invariance of intra-lesion relationship with respect to original data—despite dimensionality reduction—(3) and its prognostic power. In experiments (1) and (3) we compare the results of Dual AD-AE to those of state-of-the-art ComBat-based approaches. In experiment (2), we propose a statistical test to access the consistency of the data transformation. We evaluate our proposed models on a multi-center dataset of HL patients in order to predict response to first-line chemotherapy, demonstrating that Dual AD-AE enables building exhaustive patient representations and delivering more accurate analyses, especially when trying to exploit the predictive power of intra-tumor heterogeneity.

## Results

### Data collection

Two centers were involved in the study; inclusion criteria were age $\ge$ 16 years old, newly diagnosed stage I–IV HL and baseline [^18^F]FDG-PET/CT availability, and exclusion criteria were missing clinical/imaging/follow-up data; 128 HL patients were recruited and treated at IRCCS Humanitas Research Hospital (Institution 1), 78 at Fondazione IRCCS Istituto Nazionale dei Tumori (Institution 2). Personal information and clinical data were annotated for each patient in both hospitals and [^18^F]FDG PET/CT imaging was inspected by experienced nuclear medicine physicians. Descriptive statistics of patients are available in Supplementary Tables S1 and S2 for Institution 1 and Supplementary Tables S3 and S4 for Institution 2. Of note, number of relapsing patients was 21 over 128 (16%) in Institution 1 dataset and 17 over 78 (22%) in Institution 2 dataset. All [^18^F]FDG-avid lesions bigger than 64 voxels were segmented in each patient and radiomic features were extracted from each lesion using LIFEx software (www.lifexsoft.org^24^). A total of 1340 and 794 lesions were collected at Institution 1 and Institution 2, respectively. Information about scanners’ specification and acquisition settings is summarized in Supplementary Tables S5 and S6, while Imaging Biomarker Standardization Initiative (ISBI)-compliant standardization and data harmonization have been published elsewhere^9^. The study was approved by the local ethics committees at Institution 1 (n. 2595 on Jun16, 2020) and Institution 2 (code INT 212/20 on Sep28, 2020); given the observational retrospective design of the study, the signature of a specific informed consent was waived.

### Experimental outline

**Figure 2:**
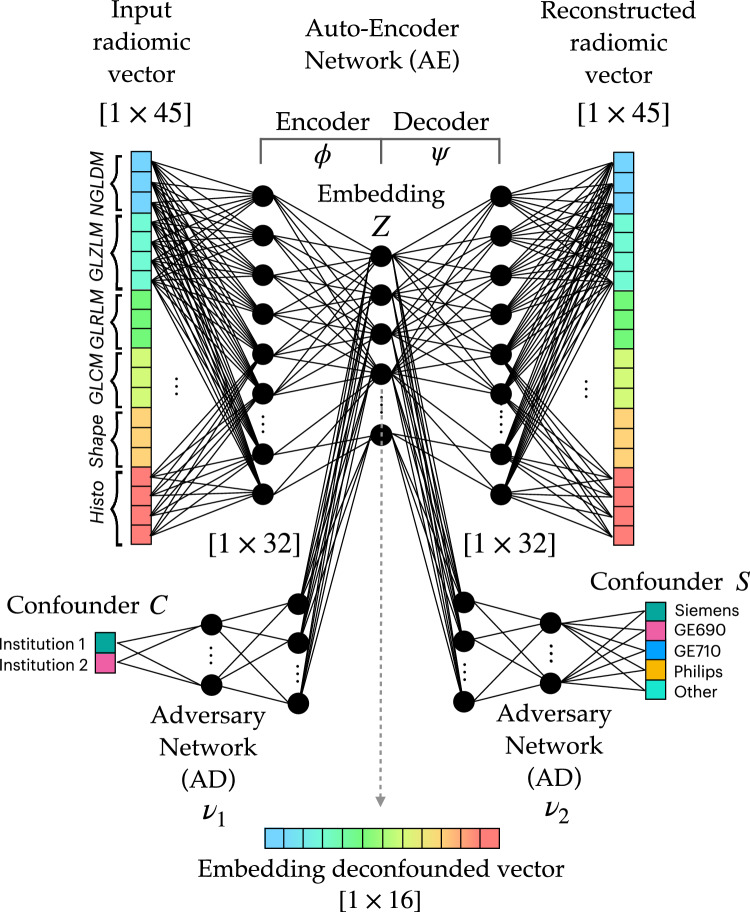
Architecture of the dual adversarial deconfounding autoencoder (AD-AE) model: the model is made of three parts: an autoencoder (encoder: $\phi$, decoder: $\psi$), an adversary branch network predicting the center confounder $(\nu _1)$ and a parallel adversary branch network predicting the scanner confounder $(\nu _2)$. The network is trained by optimizing the input reconstruction task (autoencoder loss) and the deconfusion task (adversary losses) as in Eq. (1) of “Methods”. The adversaries unlearn to predict the confounding factors, i.e. the center and the scanner.

As displayed in Fig. 2 and further explained in the “Methods”, the proposed Dual AD-AE consists of (i) an autoencoder with multiple hidden layers and (ii) two adversary branches that predict the source of data, i.e., the center and the scanner. The rationale of this method is that penalizing the prediction performance of the adversaries while jointly maximizing the reconstruction accuracy of the autoencoder will result in lesion embeddings that keep as much as possible of the original signal while discarding solely the noise introduced exogenously by the two confounders.

As mentioned in the “Introduction”, to identify a robust post-image-acquisition method to harmonize multi-lesion radiomics data from multi-center studies, one needs to consider several aspects. Indeed, to propose Dual AD-AE as better suited to the task, we performed a series of experiments on different harmonization strategies (in the following, *modalities*).

We recall that the center confounding factor relates to the hospital’s imaging facility, the clinical guidelines, and the personnel who segments and carries out the acquisition. On the other hand, the scanner confounding factor supplies information on the scanners’ specifications and reconstruction parameters. The scanner variable may ve intrinsically subordinated to the center variable, as usually different scanners are found in different centers. They may thus entail some extent of nesting nature and partially overlap in their confounding information.

For the sake of comparison with state-of-the-art approaches, we tested three major ComBat implementations, namely Combat^18^, ReComBat^20^ and OPNested Combat^22^, comparing the results to quantify the improvements of our solution. Specifically, single-factor ComBat was applied twice in cascade (in both confounders’ orders). These two ComBat-based models are namely ComBat-center-scanner and ComBat-scanner-center depending on the order of the batch-effects. The very same approach has been followed for ReComBat. OPNested Combat was instead applied once on center and scanner effects at the same time, as it was specifically developed for multi-factor effect removal.

On these models and ours, we performed three different quantitative experiments. We tested the deconfusion power of the different modalities, comparing the proposed method to the state-of-the-art models (Experiment 1). Furthermore, considering that the Dual AD-AE encompasses dimensionality reduction as part of the deconfusion process, leading to a potentially detrimental transformation of intra-lesion relationships, we developed a novel test to assess this impact quantitatively (Experiment 2). Finally, we tested and compared all modalities on their ability to keep predictive information intact. We transformed the deconfounded features of each modality into different all-lesions patients’ representations, to be fed into prognostic models and evaluated the performance of prognostic models (Experiment 3).

#### Experiment 1: checking deconfusion power

To evaluate the strength of the confounders’ effect, one can verify the predictability of the confounding variables (i.e. the center and the scanner) from the data. A high prediction performance denotes the presence of a strong confounder-related signal. Therefore, in order to quantify the effect of the deconfusion process, we compared the predictive power of cross-validated Logistic Regression models fed with the radiomics features before and after the application of the different modalities. Details on the analysis are provided in the “Methods” section. Accuracy was annotated for performance comparison through statistical tests. Table 1 shows the results for the Dual AD-AE, the two ComBat models, the two ReComBat models, and OPNested ComBat.

**Table 1:** Experiment 1 results: comparison between the performance of the Logistic Regression models for predicting the two confounding factors: the center and the scanners.

		Accuracy CENTER	Accuracy SCANNER
Baseline	Radiomics	0.8559 ± 0.0117	0.8617 ± 0.0104
Embedding	Dual AD-AE	**0.6251 ± 0.0131**	**0.3308 ± 0.0146**
ComBat	ComBat-center-scanner	**0.6220 ± 0.0137**	**0.2968 ± 0.0278**
	ComBat-scanner-center	**0.6236 ± 0.0134**	**0.3006 ± 0.0307**
ReComBat	ReComBat-center-scanner	**0.6228 ± 0.0150**	**0.3009 ± 0.0362**
	ReComBat-scanner-center	**0.6276 ± 0.0124**	**0.2997 ± 0.0359**
Opnested	Opnested (scanner-center)	**0.6239 ± 0.0118**	**0.2967 ± 0.0349**

The modalities that have been evaluated are raw radiomic data, Dual AD-AE embedding, ComBat-standardized data (both with center-scanner order and with scanner-center order), ReComBat-standardized data (both with center-scanner order and with scanner-center order) and OPNested-standardized data (with scanner-center order). The Logistics Regression models are fitted on each of these modalities, in a cross-validated fashion. Values are annotated as mean ± standard deviation. The models evaluate (1) the binary prediction of the center labels, and (2) the multi-class prediction of the scanner labels. The performances of the radiomics-based models are taken as reference, while the performances of the other modalities are analyzed in terms of decrease compared to the baseline models’ performance. Statistical tests have been performed and the models that are significantly different from radiomics are highlighted in bold.

While radiomics, as expected, scored very high in predicting both the center and the scanner (as assessed by the univariate analysis in Supplementary Table 7), our embeddings showed evidence of deconfusion, comparable to state-of-the-art benchmarks. Both the Dual AD-AE and all the Combat-based modalities aligned to the same performance, outperforming the non-deconfounded radiomic vectors. Indeed, values highlighted in bold in Table 1 correspond to non-significantly different, yet lower than radiomics, performances. All modalities were thus equally powerful at the deconfusion task. Of note, the OPNested algorithm selected scanner-center as the optimal order, thus the two models are expected to perform similarly. Additionally, the proposed Dual AD-AE model showed a smaller standard deviation of the accuracy in predicting scanner type, supporting the robustness of the model.

#### Experiment 2: cloud-shape invariance test

In multi-lesion and/or metastatic tumor settings such as HL, patients can be modeled as clouds of points^14^, where each point is defined by the radiomic vector—whether original, reduced, or deconfounded—of a lesion, and the shape of the cloud determines intra-patient tumor heterogeneity as the pairwise relationship between lesions^25^. To ensure that the predictive information of the clouds’ shape has been preserved, Dual AD-AE embeddings must keep invariance with respect to the relative positions of lesions, despite the reduced dimensionality of the resulting vectors. That is, patient-wise lesions’ rankings and pairwise lesions’ distances should hold after deconfusion, under the hypothesis that they are not independently impacted by exogenous noise. Given this assumption, to test for the cloud shape-invariance of the feature transformations, we developed a quantitative method, introducing novel metrics called Point Cloud Semantic Drift (PCSD). As further detailed in the “Methods” section, PCSD quantifies the extent of the change in peer lesions’ distance rank order within a patient. Furthermore, to define a quantitative test of hypothesis for PCSD, we estimated an empirical null distribution of the PCSD values when point clouds are randomly transformed, inducing random neighbor swaps by injecting repeatedly Gaussian noise in subsets of the embeddings. The empirical p-value of the Dual ADAE transformation was then obtained from the Empirical Cumulative Distribution Function (ECDF) of this null PCSD distribution.

**Figure 3:**
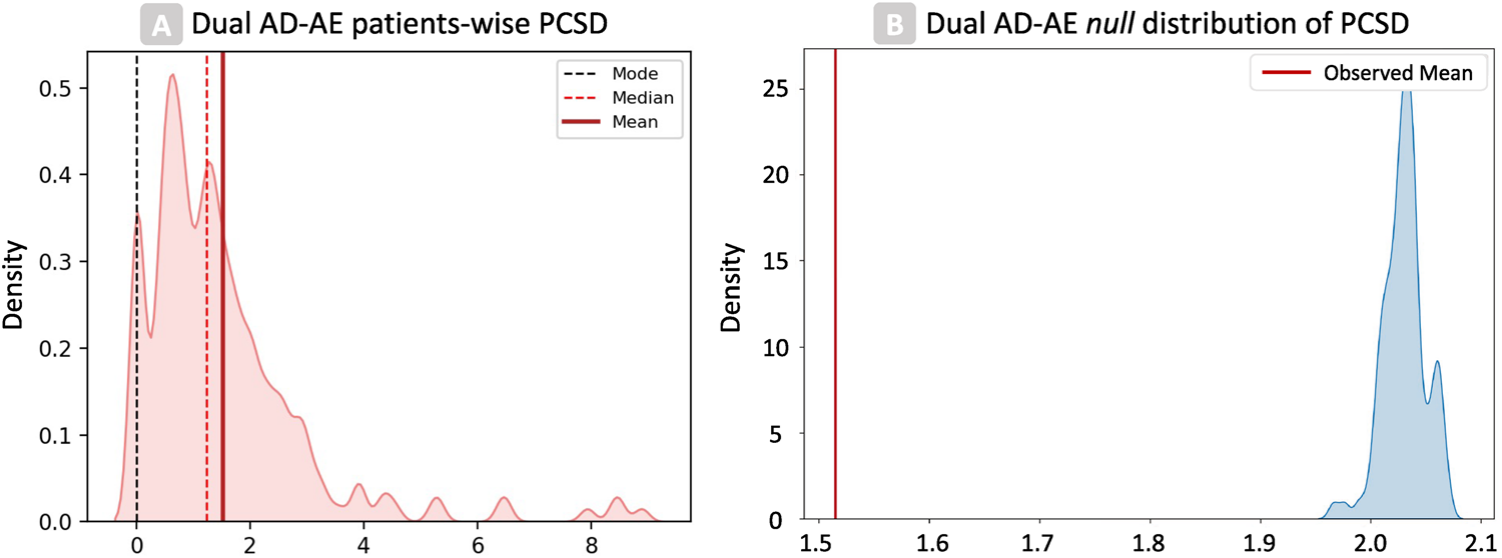
Results of patient-wise tests on PCSD for dual AD-AE embedding: (**A**) The density plot displays the overall distribution of PCSDs in the population. (**B**) The density plot shows the score of the Dual AD-AE results over a bootstrap random distribution. Fiducial values of the distribution are marked with vertical lines in the left plot and our model performances are displayed with a vertical red line in the right plot.

Figure 3 shows the results of the proposed method. The population distribution of PDSC from Dual AD-AE transformation is displayed alongside the Empirical Distribution Function (EDF) of 100 random transformations. From the visual inspection of the plots, the model produced PCSD values skewed toward zero, suggesting the shape-invariance of the clouds. Moreover, the empirical p-value was equal to zero, thus we can further sustain that Dual AD-AE successfully kept cloud-shape invariance and that the change in inter-lesion distance, which occurred during deconfusion, was significantly far from being random.

#### Experiment 3: checking prognostic power

**Figure 4:**
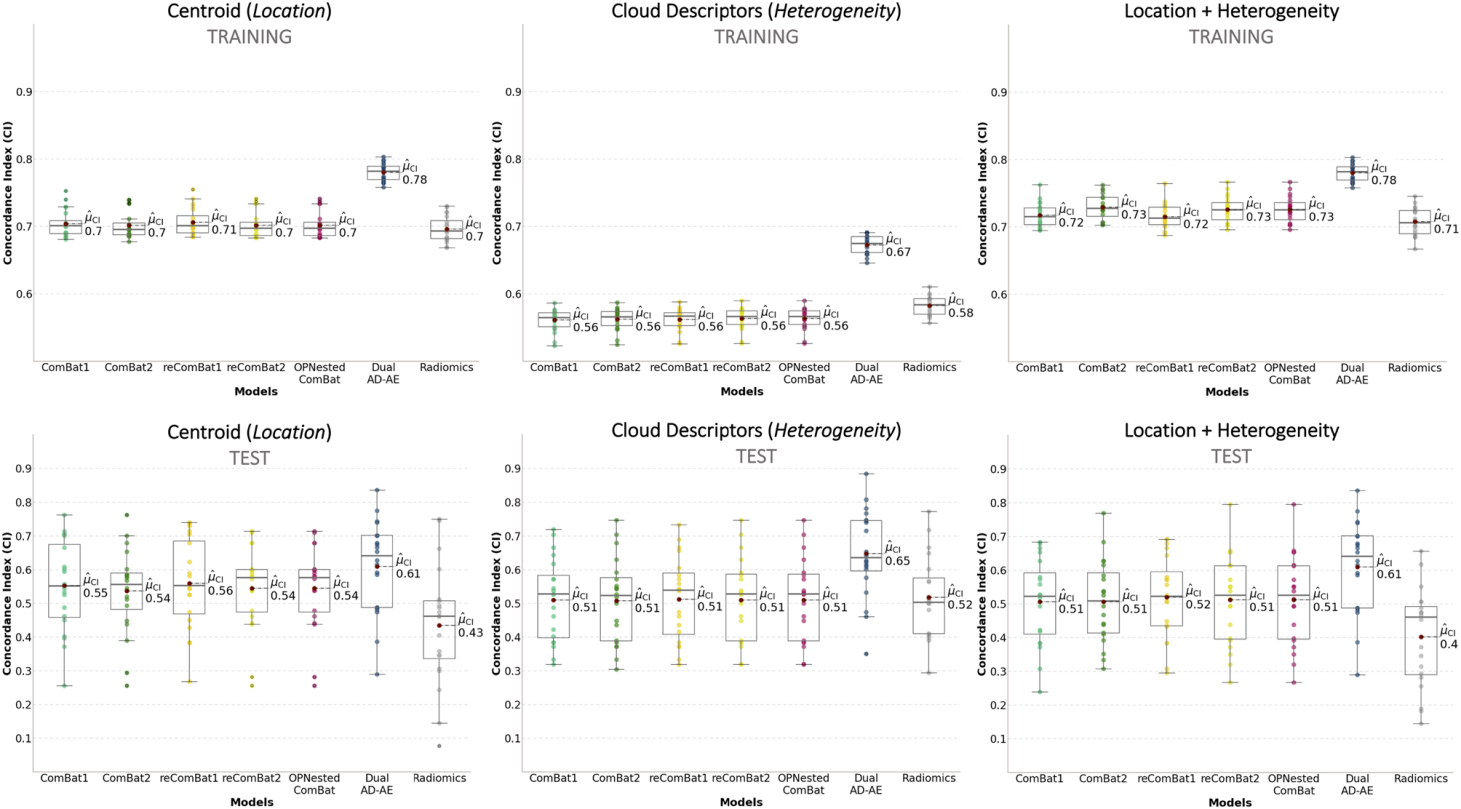
Experiment 3 results: the boxplots of the distributions of algorithms’ performances. The three different patients’ representation strategies are considered per modality and one representation is displayed per plot. The top row plots show training results while the bottom row plots show testing performances. All plots report on the y-axis the CIs of ComBat-center-scanner radiomics (light green, ComBat1 for short), ComBat-scanner-center radiomics (dark green, ComBat2 for short), ReComBat-center-scanner radiomics (dark yellow, reComBat1 for short), ReComBat-scanner-center radiomics (light yellow, reComBat2 for short), and OPNested ComBat radiomics (fuchsia), dual AD-AE embeddings (blue) and original radiomics (grey). The red dots highlight the mean CIs, which are also reported on the right of each respective boxplot.

Despite its unsupervised nature, the proposed approach aims to enable the design of exhaustive patient representations to deliver accurate analyses for treatment planning on multi-center datasets. Here we provide an example of downstream analysis where to quantify the improvement in predicting the first-line chemotherapy outcome of patients affected by HL after correcting for confounding factors. To do this, we resorted to the use of three patients’ representations, encompassing both separately and jointly the location and the shape of the point clouds. In particular, we represented each patient as a point cloud and defined (1) a mean vector of all lesions of each patient (i.e. the centroid of the cloud), (2) a set of topological indexes describing the structure/shape of the clouds (i.e. the mean and the standard deviation of the pairwise distances between lesions and the mean and the standard deviation of the distances between lesions and the cloud centroid) and (3) a representation including both the centroid vector and the cloud describing indexes. Further details are provided in the “Methods” section. These three representations were constructed from original radiomics features and their deconfounded versions with all the considered modalities (Dual AD-AE embeddings; ComBat-, ReComBat- and OPNested ComBat-transformed radiomics). Each of them was fed into a Cox proportional hazard model^26^ to predict the time-varying response to therapy. Of note, vectors derived from the state-of-the-art algorithms needed to be reduced by PCA prior to being input into any model. The significance of the prognostic power of imaging information was assessed in terms of the Concordance Index (CI^27^). Performances on both the training and testing phases were produced by repeated sampling of 20 independent data splits. Supplementary Table S7 reports the means and standard deviations of the trials. For visual reference, Figure 4 displays the boxplots of the distributions of the performance indexes of the modalities, grouped by patients’ representation strategy and deconfusion approach. Pairwise tests were performed between settings to be compared and can be appreciated in Table 2.

**Table 2:** Experiment 3 results: p-values of the tests comparing different survival models.

Comparison wrt	Centroid		Cloud description		Centroid + cloud description	
	P-value (train)	P-value (test)	P-value (train)	P-value (test)	P-value (train)	P-value (test)
Radiomics model	**<< 0.001**	**<< 0.001**	**<< 0.001**	**0.0001**	**<< 0.001**	**<< 0.001**
ComBat-center-scanner model	**<< 0.001**	0.1065	**<< 0.001**	**0.0003**	**<< 0.001**	**0.0230**
ComBat-scanner-center model	**<< 0.001**	**0.0473**	**<< 0.001**	**0.0002**	**<< 0.001**	**0.0160**
ReComBat-center-scanner model	**<< 0.001**	0.1381	**<< 0.001**	**0.0005**	**<< 0.001**	**0.0368**
ReComBat-scanner-center model	**<< 0.001**	0.0676	**<< 0.001**	**0.0003**	**<< 0.001**	**0.0274**
OPNested ComBat model	**<< 0.001**	0.0676	**<< 0.001**	**0.0003**	**<< 0.001**	**0.0274**

The Dual AD-AE model is considered according to its three different patient representations: (1) the patient is described by the centroid of their point cloud, (2) the patient is described by the topological characteristics of their point cloud and (3) the patient is described by both the centroid and the topological characteristics of their point cloud. These are compared with the radiomics-based models, the ComBat-based models, the ReComBat-based models, and with the OPNested-based models. Comparisons are made upon the same patient representation: for instance, the Dual AD-AE model fed with centroid representation is compared to the other modalities which were fed with centroid representation as well, and so on. Significant values are highlighted in bold.

As displayed in Table 2, the model performance of the Dual AD-AE modality was significantly higher than radiomics’, suggesting how the deconfusion step does also benefit the prediction and the signal-to-noise ratio. Of note, the patient representations including cloud topology descriptors (i.e. when using heterogeneity as a predictor) always achieved better performance than the benchmarks, being the most predictive and generalizable (i.e. test set performance) overall. From what centroid representation is concerned, ComBat-center-scanner, ReComBat-center-scanner, ReComBat-scanner-center, and OPNested ComBat scored similarly yet worse with respect to Dual AD-AE. We remind that the OPNested algorithm implemented the same sequence of ComBat-scanner-center, however, this latter model had lower performance, being significantly outperformed by our model.

### Alternative to deconfusion: frailty Cox proportional hazards model

Deconfusion methods ultimately allow the effective modeling of patients’ representations across scanners and centers. However, instead of removing the confounding factor, an alternative yet well-established approach to model multi-source samples (i.e. multi-center/multi-scanner data, where we have dependence within groups) is the explicit modeling of the group-specific variability within the prediction model. For time-to-event data, this can be done via the frailty Cox proportional hazards model^28^, which estimates center and/or scanner random effects together with the baseline hazard function. To verify whether this approach would make the deconfusion step irrelevant, the centroid representation derived from the raw radiomics features was reduced by PCA and fed into a frailty Cox proportional hazard model with center-specific and scanner-specific random intercepts. Unfortunately, this test dramatically failed due to a lack of model convergence. This result is motivated by the small sample size of the data at hand, combined with the high dimensionality of the radiomic variable (even after PCA) and the large number of censored patients, which did not allow the model to properly estimate the effects’ parameters neither on the training sets, on the testing sets, nor on the dataset as a whole.

## Discussion

In this work, we developed a deconfusion algorithm to harmonize multi-center imaging data, with a particular focus on multi-lesion/metastatic cancers, like Hodgkin Lymphoma. The Dual AD-AE model performed dimensionality reduction of radiomic features while removing center- and scanner-related information simultaneously. The proposed approach was trained on a dataset of Hodgkin Lymphoma patients from two centers and outperformed the state-of-the-art methods in the task of radiomic features harmonization, leading to higher prediction of response to first-line chemotherapy.

Three experiments were performed to evaluate the model’s properties, raising some major points of discussion. First, the deconfounding power of the Dual AD-AE was granted. In fact, the accuracy of Logistic Regression models predicting the scanner and the center target variable sensibly decreased after deconfusion. The Dual AD-AE demonstrated a comparable deconfusion power with respect to ComBat-based models, showing no statistical differences in cross-validation. However, removing both confounding factors at the same time may uncover and discard inter-confounder relationships which may contribute to undesirable noise in the signal. Interestingly, the standard deviation of the accuracy of the Dual AD-AE model in predicting the scanner type was lower than other models, suggesting the robustness and stability of the proposed model. The ComBat (and ReComBat) algorithm applied twice showed variable results when changing the order of application. This inconsistency is not surprising, as it motivated the development of OPNested ComBat in the first place^21,22^. In fact, despite the slight algorithmic differences between ComBat and OPNested, OPNested performed very similarly to ComBat-scanner-center.

Additionally, as the context of multi-lesion/metastatic data may benefit from the exploitation of intra-tumor heterogeneity as predictive information, we designed a novel metric (i.e. PCSD) and an associated empirical test to quantify the impact of the Dual AD-AE deconfusion and dimensionality reduction on intra-lesion relationships shaping the spatial conformation of patients’ point clouds. Overall, the Dual AD-AE resulted in a significantly low PCSD value, rejecting the null hypothesis of no correlation between the original (raw) and the deconfounded clouds of lesions. On one hand, this was expected and desired as lesions of one patient share both the same center and scanner variability. That is, noise can be considered constant within a single patient and the relationship among peer lesions should in principle not be spoiled by center and scanner deconfusion. On the other hand, it might be possible that minor shifts could be appreciated in specific lesions, especially where massive non-linear transformations were needed to properly clear the data. This might be true for some patients lying on the far-right tail of the PCSD distribution. As proved by the test, such results do not translate into a detrimental data transformation, rather they show that a trade-off between deconfusion and cloud-shape invariance has to be tuned and rigorously assessed. On purpose, the PCSD metric can be exploited to highlight the presence of such additional sources of latent and interactive noise, that once removed would release the true predictive power of intra-lesion heterogeneity.

This point was further validated in the third experiment presented in this work, where we assessed the increase in the prognostic power of the deconfounded representation of patients in terms of response to therapy, against ComBat-based alternative approaches. In principle, a proper deconfusion allows the shape and location of the point clouds coming from different sources to be meaningfully compared. Thus, one can expect that predictive models built on these clouds’ representation, that is lesions’ characteristics and intra-tumor heterogeneity, benefit from the deconfusion process. In fact, Dual AD-AE embeddings showed significant improvements with respect to the baseline and the benchmarks, even though the gap between training performance and testing performance remains large and would necessitate some prevention strategies to overfitting. The results however testify how the proposed model can identify and remove the complex and potentially non-linear portion of confounders’ noise that the competitors ignore. Moreover, it demonstrates the relevance of removing all confounders simultaneously when in presence of multiple factors of variability in the data.

A further particularly relevant result was the difference in performance when using heterogeneity (i.e. cloud describing indexes) as a predictor. While this cloud shape representation was merely a simple proof-of-concept example, Dual AD-AE embedding was seen to allow for a much better prediction than the baseline model and competitors. Conversely, ComBat-based and ReComBat-based benchmarks seemed to corrupt the heterogeneity signal to the point of achieving lower CI than the original radiomics features during training, and they grant just a very limited performance increase during testing. Additionally, to the best of our knowledge, none of the previous studies comparing deconfusion algorithms for radiomics data^15–17^ evaluated their impact on the predictive power of groups of lesions. Here, our proposed approach was the only deconfounding algorithm truly releasing the predictive power of heterogeneity, which became the most generalizable predictor.

This finding leads to two relevant considerations. Clinically speaking, it supports the hypothesis that intra-lesion heterogeneity does carry predictive information, once properly corrected for linear and non-linear confounders. Technically, it endorses the use of a more complex non-linear model like the AD-AE, that can uncover and remove explicit and latent types of noise effectively. Although not explicitly enforcing inter-lesion relationships consistency in the model we propose, so that it could be in principle applied as-is to single-lesion data, this result testifies in favor of its application (as opposed to the state-of-the-art) to contexts in which heterogeneity information is crucial for prediction.

Of course, training complex, non-linear, and heavily parametrized models such as the Dual AD-AE has higher computational, time, and memory demands compared to the simpler ComBat-based methods. Nevertheless, the latter algorithms rely on Gaussian distribution assumptions for estimating the parametric definitions of the statistical moments across batches (i.e. the mean and the variance across centers or scanners), prior to standardization. However, this strong hypothesis of underlying data structure may not always be appropriate for radiomics data, leading to underpowered and biased transformations. Conversely, we proposed a non-parametric algorithm removing linear and non-linear confounder-induced noise without any prior assumption. Furthermore, the Dual AD-AE was the only method that dealt with two confounders simultaneously. This permitted to reduce the risk of ignoring the portion of noise induced by center and scanner interactions (for instance, if one center uses way more frequently a set of parameters for a specific scanner, compared to other centers). Moreover, thanks to its modular nature, one could easily extend the model to adversarially predict—that is, unlearn—more than two confounders. In fact, additional branches could be added, and the overall loss might be updated with the maximization of the corresponding accuracies. Further, the weighting parameters $\lambda _i$ (with *i* being the number of adversary branches) enable defining the impact of each confounder, rebalancing the expected (or measured) relative effect of noising factors on the data. Both these aspects could hardly be integrated into the ComBat approach. Finally, as opposed to ComBat-based methods, Dual AD-AE performs dimensionality reduction together with cleaning of the embeddings. While this may affect the interpretability of the deconfounded data, we argue that radiomics features are not easily interpretable per se, and they usually need a dimensionality reduction (such as PCA) before modeling, as they are highly collinear.

As a final remark, disregarding the deconfounding algorithm employed, the two-step pipeline of removing confounding effects and then analyzing the corrected data has raised several critiques^29,30^. Oppositely, the most sponsored solution when the confounder information is available is including it within the final prediction model. Nevertheless, we have shown in our last tentative experiment how a frailty CoxPH model (even if with only one confounder) does not converge when the sample size is small and the number of censored patients is high. This is quite common in multi-center studies of rare diseases.

A limitation of the present study is the lack of further data to test our proposed approach. However, no additional comparable data was available to the authors at the time of writing. Nevertheless, we believe that the comprehensive tests and benchmark studies performed on these cohorts represent a valuable proof-of-concept of the method’s potential. Moreover, data provenance may behave as a bias in our experiments. Yet, despite the data originated from two geographically close hospitals with standardized procedures and consistent image acquisition and feature extraction protocols, inherent heterogeneity and discrepancies in data values persisted, supporting the objective of implementing a feature-level harmonization^31^. This emphasizes the need for a comprehensive approach that combines image standardization, post-processing, and harmonization models to eliminate batch effects and achieve data consistency.

In conclusion, we provided a modular and effective approach for harmonizing imaging data coming from different sources. We proved that our approach could efficiently correct for multiple batch-related differences so that data appear as if they were acquired under a common set of conditions. This translates to higher prognostic performances, above all for what regards intra-tumor heterogeneity of multi-lesions/metastatic cancers. As it is well known that NN models such as the Dual AD-AE can benefit from Transfer Learning^32^ to aid the problem of suboptimal and/or overfitting parameters when training data is limited, we provide a tutorial to apply our method to new data, available on GitHub (https://github.com/LaraCavinato/Dual-ADAE). We currently share the weights of our pre-trained network on this study’s cohorts. Researchers might thus decide to use such weights to pre-train their Dual AD-AE model, “borrowing” information from additional samples without privacy concerns. This model-sharing framework could be pushed forward with the contribution of the scientific community sharing their fine-tuned parameters, paving the way for a virtuous cycle of open science. Insightful knowledge could be thus derived from more exhaustive models to optimally impact the clinical practice.

## Methods

### Dual adversarial deconfounding autoencoders

Dual adversarial deconfounding autoencoder (Dual AD-AE) jointly tackles the denoising from both center- and scanner-related information. The architecture of the Dual AD-AE is described in Fig. 2. The network consists of two parts: one autoencoder and adversary branches. The autoencoder takes as input the radiomic vector associated with a lesion and performs the dimensionality reduction. It is made of one input layer (number of input nodes: $[1 \times 45]$) two hidden layers (number of first hidden layer nodes: $[1 \times 32]$, number of second hidden layer nodes: $[1 \times 16]$), and one output layer (number of output nodes: $[1 \times 45]$). The autoencoder represents the backbone of the model and, from its deepest layer, two adversary networks branch out for center and scanner predictions. Both adversary networks are made of two hidden layers (dimensions of the first hidden layer and the second hidden layer are $[1 \times 50]$ and $[1 \times 50]$ respectively) and one output layer ($[1 \times 2]$ for center prediction and $[1 \times 5]$ for the branch predicting the scanners).

The loss is then made of three terms, where the reconstruction error, the accuracy of the center classification, and the accuracy of the scanner classification sum up as in Eq. (1):

1 $$\begin{aligned} \min _{T}(\phi ,\psi ,\nu ) E [ |x - g_{\psi } (f_{\phi } (x))|_2^2 - \lambda _1 L (h_{\nu _1} (x), c ) - \lambda _2 L (h_{\nu _2} (x), s )] \end{aligned}$$

where $\nu _1$ is the center adversary branch, $\nu _2$ is the scanner adversary branch, $\lambda _1$ and $\lambda _2$ are weighting parameters and *c* and *s* are the true labels for center and scanner respectively. Of note, weighting parameters can be tuned to tailor the importance of the tasks to be optimized. For instance, one could prioritize one confounding factor rather than the other, having a priori information about the latent variability of the specific case study data.

In our setting, hyperparameters were tuned according to grid search. The number of layers, the number of nodes, and weighting parameters were optimized based on the reconstruction error. The number of epochs was optimized according to early stopping strategy^33^, i.e., iterations were stopped when no relevant improvements of the validation loss were recorded. The batch size was set to 128 and $\lambda _1 = \lambda _2 = 1$.

### Benchmark state-of-the-art methods

Among the methods proposed in the literature for imaging harmonization, ComBat has been repeatedly elected as the best approach such that different implementations and further improvements have been proposed in the last years.

ComBat was originally proposed by Johnson and Rabinovic^18^ for removing the batch-effect seen in genetics microarray analysis. The harmonization method consists of standardizing each batch according to its mean and variance. Specifically, the correction takes place at a specific location and scale (*L*/*S*), wherein the batch-related error is supposed to be present. *L*/*S* model states that the value *Y* for feature *f* from a sample *j* in a batch *i* follows the following formulation:

2 $$\begin{aligned} Y_{ijf} = a_f + X \beta _f + \gamma _{if} + \delta _{if} \varepsilon _{ijf} \end{aligned}$$

where $a_f$ is the feature value, behaving as intercept; *X* is the design matrix and $\beta _f$ is the features coefficients such that $X \beta _f$ is the observed variability; $\gamma _{if}$ and $\delta _{if}$ are the additive and multiplicative batch effects respectively and $\varepsilon _{ijf}$ the standard error. Accordingly, $\gamma _{if}$ and $\delta _{if}$ can be estimated (either in parametric and non-parametric ways) from data, and $Y_{ijf}$ can be corrected as:

3 $$\begin{aligned} Y_{ijf}^* = \frac{Y_{ijf} - \hat{a}_f - X \hat{\beta }_f - \hat{\gamma }_if}{\delta _{if}} + \hat{a}_f + X \hat{\beta }_f \end{aligned}$$

One of the main advantages of ComBat is being effective even with small batch sizes. Being $A = \tilde{X}^T \tilde{X}$ positive-definite, the optimization problem is strictly convex. However, when *A* happens to be singular the regression estimation does not exist and, if the system is underdetermined, ComBat is not guaranteed to bring out a unique solution. For this reason, Adamer et al.^20^ proposed a regularized solution of ComBat (ReComBat) computationally more efficient to facilitate the large-scale harmonization of data.

As to compare our method with the state of the art, we applied both ComBat and ReComBat models to our case study. We employed different pipelines to test their performance from different perspectives. ComBat was used for deconfounding the imaging data from the center and scanner information. The two ComBat models were applied in cascade to the data: (1) one label was used as a batch effect to be removed and (2) the obtained denoised vector was further deconfounded by the effect of the other label. We followed two different orders, namely ComBat-center-scanner and ComBat-scanner-center. The very same procedure was investigated by employing ReComBat implementation. Two different pipelines were thus derived, namely ReComBat-center-scanner and ReComBat-scanner-center.

As a matter of fact, applying ComBat or ReComBat in cascade to capture and remove the linear variability from more than one confounding factor may cause instabilities depending on the specific order of the harmonization steps. Very recently, Horng et al.^21,22^ proposed an optimized procedure for sequentially harmonizing data from multiple batch effects, namely OPNested ComBat. Besides ComBat and ReComBat, we included OPNested as a benchmark model, to be tested in both deconfusion and predictive powers. On one hand, OPNested can show a more effective standardization procedure to compensate for the heterogeneity of diverse data sources, improving the generalization abilities of imaging data. On the other hand, higher harmonization performance might not imply higher predictive performance as it yet remains to be investigated whether and which latent factors have to be removed or smoothed.

### Evaluating point-cloud shape consistency

We defined and implemented a novel approach to test the point-cloud shape consistency across transformation: the Point Cloud Semantic Drift (PCSD).

Before defining PCSD, let us introduce some necessary notation. Let $M_i(1) \ldots M_i (K)$ be the scores associated with the ordered list $L_i$, where $M_i (1)$ is the best score, $M_i (2)$ is the second best, and so on. The best score can be the largest or the smallest depending on the context. Let $r^{L_i (A)}$ be the rank of *A* in the list $L_i$ if element *A* is within the top *k* elements, and be it equal to $k+1$ otherwise; $r^{\delta } (A)$ is defined likewise for a different list $\delta$. The Spearman’s footrule distance between $L_i$ and any ordered list $\delta$ can be defined as:

4 $$\begin{aligned} S(\delta ,L_i) = \sum _{t \in L_i \cup \delta } | r^{\delta } (t) - r^{L_i} (t)|. \end{aligned}$$

Equation (4) is the sum of the absolute differences between the ranks of all the unique elements of the union of the two ordered lists. The smaller the value of the metric, the more similar the lists. To compute the Point Semantic Drift (PSD) for an arbitrary point *t*, we exploit a weighted version of *S*. We estimate the PSD as the weighted change in neighbor rankings, according to Eq. (5).

5 $$\begin{aligned} PSD(\delta ,L_i) = \sum _{t \in L_i \cup \delta } |M(r^{\delta } (t)) - M(r^{L_i} (t))| \times |r^{\delta } (t) - r^{L_i} (t)|. \end{aligned}$$

PSD is the sum of penalties for moving an arbitrary element (data point) *t* of the list $L_i$ from a position $r^{\delta }(t)$ to another position $r^{L_i}(t)$ within the same list (second term of the product) adjusted by the difference in scores between the two positions (first term). $M(r^{\delta } (t))$ and $M(r^{L_i} (t))$ are the normalized distances between *t* and all other points in the cloud, respectively after and prior to any transformation. This weighting scheme penalizes more the changes in the positions of very distant points, than the neighboring shifts of observations lying close in the original cloud. That is, higher weights are assigned to swaps between close-by and far-distant points, compared to changes among close neighbors. Such information can deeply inform the deconfusion-invariance trade-off.

Once computed the PSD for each point in the cloud *C*, the Point Cloud Semantic Drift is estimated as the average PSD$_k$ of the *K* points in *C*:

6 $$\begin{aligned} PCSD = \frac{1}{K} \sum _{k=1}^{K} PSD_k \end{aligned}$$

where *K* is the number of lesions in the patient under consideration.

In our setting, $L_i$ corresponds to the set of lesions of patient *i* as described by the raw radiomic features (original set); $\delta$ corresponds to the set of lesions described by the transformed features after deconfusion (e.g. Dual AD-AE mode). PCSD thus accesses and quantifies the invariance of each cloud (patient) to the data transformation process.

Given that PCSD can take on values ranging from 0 to infinity, we need to establish a suitable test to assess the significance of the results obtained from our deconfounded point clouds. To accomplish this, we can build a null distribution of PCSD values (PCSD$_{null}$) which serves as an upper bound for the drift. That is, it represents the change in the cloud’s shape that would occur if an arbitrary embedding function was employed, completely disregarding the initial data structure. Operationally, we randomly transform the original cloud by adding a random Gaussian noise with mean $\mu =0$ and variance $\sigma = 3$ to a different subset of the lesions’ vectors. We do this iteratively 100 times, computing the PCSD each time. Upon these values, we build the Empirical Cumulative Distribution Function. If the true PCSD value obtained from our deconfounded embeddings falls within the limits of the left tail of this empirical null distribution, significant evidence is obtained on the ability of our algorithm to maintain the original cloud structure. The empirical p-value is computed from the Empirical Cumulative Distribution Function by computing the ratio between the number of trials where the PCSD is lower than the computed real value and the total number of trials (100).

### Experiments’ implementation details

Three tests have been implemented to test for (1) deconfusion power, (2) transformation consistency, and (3) predictive power of the proposed algorithm compared to current literature models.

The deconfusion power has been tested by predicting the confounder(s) using the features under analysis. We employed a cross-validated Logistic Regression model, with 100 trials and replacement. Testing accuracy was annotated in each trial to compute the mean trend and the standard deviation of the performance of each modality. Additionally, to compare the performance of the models, given the normality of the data, we used a two-sided parametric t-test for paired samples and evaluated the improvements of the different harmonization strategies with respect to the pure radiomics description.

The point cloud shape consistency between radiomics data and transformed data was evaluated as described above. The PCSD was computed for each patient and a population test for testing the transformation consistency was carried out in the context of the Dual AD-AE.

Lastly, the predictive power of the imaging features has been evaluated with Cox proportional hazard survival models in a cross-validation fashion. Three patient representation strategies were implemented to summarize multi-lesion information in a single vector object to be properly fed into the models. First, the centroid of each patient’s point cloud was computed as the mean profile of peer lesions belonging to them (“centroid representation”). Then, as a second patient representation, only the distribution of the lesions over the space was described and used as model input. For each patient, we computed the pairwise distances between all lesions in the patient and we calculated the mean and the standard deviation as an index for lesions’ variability. Moreover, we took the distances between every lesion of the patient and their centroid and kept the average and the standard deviation of these distances to quantify the lesions’ spreading from their center. Thus, the four indexes were exploited as “point cloud description representation” to be fed into the survival model. Finally, the two above mentioned representations were merged in a “complete representation” of the patient encompassing both the mean disease profile of patients and the variability of their lesions. For each of the modalities under testing, the three representations were computed and fed into Cox models. Additionally, raw radiomic, ComBat- and ReComBat-based standardized radiomic features were reduced using PCA. To result in a dimensionality comparable to the embeddings, we kept the first sixteen principal components, accounting for at least 90% of the variability. Training and testing sets are repeatedly split multiple times (20 splits) and c-index scores were reported to assess the improvements that the harmonization step brings in terms of prognostic power. To do this, given the normality of the data, one-sided parametric t-tests for paired samples were employed to establish the optimal harmonization strategy. Specifically, the Dual AD-AE embeddings’ performance was compared to ComBat-center-scanner, ComBat-scanner-center, ReComBat-center-scanner, ReComBat-scanner-center, and OPNested ComBat.

### Supplementary Information

Supplementary Tables.

## Data Availability

The data that support the findings of this study are available from IRCCS Humanitas Research Hospital and Fondazione IRCCS Istituto Nazionale dei Tumori but restrictions apply to the availability of these data, which were used under license for the current study, and so are not publicly available. Data are however available from the authors upon reasonable request and with permission of both IRCCS Humanitas Research Hospital and Fondazione IRCCS Istituto Nazionale dei Tumori. Code is available at this link https://github.com/LaraCavinato/Dual-ADAE.
