# Supplementary material for: Dual adversarial deconfounding autoencoder for joint batch-effects removal from multi-center and multi-scanner radiomics data
Source: Sci Rep. 2023 Nov 1;13:18857. doi: 10.1038/s41598-023-45983-7 (PMC10620174; doi:10.1038/s41598-023-45983-7)
Supplement: Supplementary file 1 — Supplementary Tables. [file 41598_2023_45983_MOESM1_ESM.pdf]

Dual Adversarial Deconfounding Autoencoder for joint batch-effects removal from multi-center and multi-scanner radiomics data: Supplementary materials

Lara Cavinato[1,+,*], Michela Carlotta Massi[2,+], Martina Sollini[3,4], Margarita Kirienko[5], and Francesca Ieva[1,2]

[1]Department of Mathematics, Politecnico di Milano, Via Bonardi, 9, Milan, 20133, Italy
[2]Health Data Science Center, Human Technopole, Viale Rita Levi-Montalcini, 1, Milan, 20157, Italy
[3]Department of Biomedical Sciences, Humanitas University, Via Rita Levi Montalcini, 4, Pieve Emanuele, 20090, Italy
[4]Department of Nuclear Medicine, IRCCS Humanitas Research Hospital, Via Alessandro Manzoni, 56, Rozzano, 20089, Italy
[5]Fondazione IRCCS Istituto Nazionale dei Tumori, Via Giacomo Venezian, 1, Milan, 20133, Italy
[*]lara.cavinato@polimi.it
[+]these authors contributed equally to this work

ABSTRACT

Medical imaging represents the primary tool for investigating and monitoring several diseases, including cancer. The advances in quantitative image analysis have developed towards the extraction of biomarkers able to support clinical decisions. To produce robust results, multi-center studies are often set up. However, the imaging information must be denoised from confounding factors – known as batch-effect – like scanner-specific and center-specific influences. Moreover, in non-solid cancers, like lymphomas, effective biomarkers require an imaging-based representation of the disease that accounts for its multi-site spreading over the patient's body. In this work, we address the dual-factor deconfusion problem and we propose a deconfusion algorithm to harmonize the imaging information of patients affected by Hodgkin Lymphoma in a multi-center setting. We show that the proposed model successfully denoises data from domain-specific variability (p-value<0.001) while it coherently preserves the spatial relationship between imaging descriptions of peer lesions (p-value=0), which is a strong prognostic biomarker for tumor heterogeneity assessment. This harmonization step allows to significantly improve the performance in prognostic models with respect to state-of-the-art methods, enabling building exhaustive patient representations and delivering more accurate analyses (p-values<0.001 in training, p-values<0.05 in testing). This work lays the groundwork for performing large-scale and reproducible analyses on multi-center data that are urgently needed to convey the translation of imaging-based biomarkers into the clinical practice as effective prognostic tools. The code is available on GitHub at this link.

1 Patients' characteristics

Table S 1. Patients' characteristics in Institution 1: variables are divided into categorical (number, percentage on the total) and numerical (mean, standard deviation). In the first group, they are listed the stage (four statuses), the sex (female F and male M), the presence of B symptoms like fever, sweats, weight loss (yes Y and no N), status of the disease (extranodal disease: yes Y and no N; bone disease: yes Y and no N), administration of radiotherapy (yes Y and no N), the outcome of interim PET (iPET, Deauville Score DS of the PET), end of treatment PET (EOT PET, Douville Score DS of the PET). Statistics are stratified by the treatment response, thus patients are divided into responders and non-responders.

Categorical variables – N (%)		Responders (N=107)	Non-responders (N=21)
**Stage**	I	9 (8%)	0 (0%)
	II	57 (53%)	11 (52%)
	III	12 (11%)	2 (10%)
	IV	30 (28%)	8 (38%)
**Sex**	F	62 (58%)	14 (67%)
	M	45 (42%)	7 (33%)
**B symptoms**	N	60 (56%)	7 (33%)
	Y	47 (44%)	14 (67%)
**Extranodal disease**	N	74 (69%)	11 (52%)
	Y	33 (31%)	10 (48%)
**Bone disease**	N	80 (75%)	18 (86%)
	Y	27 (25%)	3 (14%)
**Radiotherapy**	N	38 (35%)	17 (81%)
	Y	69 (65%)	4 (19%)
**iPET**	DS1	82 (77%)	10 (48%)
	DS2	12 (11%)	2 (9%)
	DS3	11 (10%)	1 (5%)
	DS4	2 (2%)	5 (24%)
	DS5	0 (0%)	3 (14%)
**PET EOT**	DS1	77 (72%)	13 (62%)
	DS2	11 (10%)	3 (14%)
	DS3	10 (9%)	1 (5%)
	DS4	3 (3%)	1 (5%)
	DS5	6 (6%)	3 (14%)

Table S 2. Patients' characteristics in Institution 1: variables are divided into categorical (number, percentage on the total) and numerical (mean, standard deviation). Among the numerical variables, there are age, number of nodal lesions of the patients, number of extranodal lesions of the patients, and time to relapse (for censored patients, the time to last follow-up is taken). Statistics are stratified by the treatment response, thus patients are divided into responders and non-responders.

Numerical variables – mean (std deviation)		
Age	39.252 (15.875)	40.143 (15.963)
# Nodal lesions	6.673 (4.813)	6.619 (6.184)
# Extranodal lesions	1.916 (5.750)	3.857 (10.256)
Time to relapse/follow-up [days]	1126.97 (704.94)	358.86 (322.854)

Table S 3. Patients' characteristics in Institution 2: variables are divided into categorical (number, percentage on the total) and numerical (mean, standard deviation). In the first group, they are listed the stage (four statuses), the sex (female F and male M), the presence of B symptoms like fever, sweats, weight loss (yes Y and no N), status of the disease (extranodal disease: yes Y and no N; bone disease: yes Y and no N), administration of radiotherapy (yes Y and no N), the outcome of interim PET (iPET, positive or negative), end of treatment PET (EOT PET, positive or negative). Statistics are stratified by the treatment response, thus patients are divided into responders and non-responders.

**Categorical variables – N (%)**		**Responders (N=59)**	**Non-responders (N=17)**
**Stage**	I	1 (2%)	0 (0%)
	II	31 (52%)	4 (23%)
	III	6 (10%)	1 (6%)
	IV	21 (36%)	12 (71%)
**Sex**	F	34 (58%)	8 (47%)
	M	25 (42%)	9 (53%)
**B symptoms**	N	35 (59%)	4 (23%)
	Y	24 (41%)	13 (77%)
**Extranodal disease**	N	39 (65%)	7 (41%)
	Y	20 (45%)	10 (59%)
**Bone disease**	N	44 (75%)	13 (77%)
	Y	15 (25%)	4 (23%)
**Radiotherapy**	N	20 (45%)	14 (82%)
	Y	39 (65%)	3 (18%)
**iPET**	Negative	55 (93%)	8 (47%)
	Positive	4 (7%)	9 (53%)
**PET EOT**	Negative	59 (100%)	0 (0%)
	Positive	0 (0%)	17 (100%)

Table S 4. Patients' characteristics in Institution 2: variables are divided into categorical (number, percentage on the total) and numerical (mean, standard deviation). Among the numerical variables, there are age, number of nodal lesions of the patients, number of extranodal lesions of the patients, and time to relapse (for censored patients, the time to last follow-up is taken). Statistics are stratified by the treatment response, thus patients are divided into responders and non-responders.

**Numerical variables – mean (std deviation)**		
Age	36.478 (13.915)	42.867 (17.868)
# Nodal lesions	7.271 (5.499)	9.706 (6.362)
# Extranodal lesions	2.288 (5.789)	3.706 (7.355)
Time to relapse/follow-up [days]	1105.72 (546.490)	257.59 (167.17)

2 Scanners' specifications

Table S 5. Image acquisition protocols and scanner specification in Institution 1: 85 patients were scanned with Siemens Biograph scanner; 51 patients were scanned with General Electric Discovery 690 scanner; 5 were scanned with other unspecified scanners.

Institution 1	Biograph – Siemens		Discovery 690 – General Electric	
	PET	CT	PET	CT
Min/bed position (static/dynamic)	2.5 (static)	–	2 (static)	–
Crystal	LSO	–	LYSO	–
Reconstruction	Iterative	–	Iterative, TOF Sharp IR	–
Attenuation correction	On CT data	–	On CT data	–
Matrix (pixels)	128×128	512×512	256×256	512×512
Resolution (mm)	5.3×5.3	0.98×0.98	2.73×2.73	1.37×1.37
Slice thickness (mm)	2.0	4.0	3.27	3.27
Slices	–	6	–	64

Table S 6. Image acquisition protocols and scanner specification in Institution 2: 34 patients were scanned with General Electric Discovery 710 scanner; 38 patients were scanned with Philips Gemini scanner; 1 patient was scanned with other unspecified scanners.

Institution 2	Discovery 710 – General Electric		Gemini - Philips	
	PET	CT	PET	CT
Min/bed position (static/dynamic)	2 (static)	–	2 (static)	–
Crystal	LYSO	–	BGO	–
Reconstruction	VPFX	–	Iterative	–
Attenuation correction	On CT data	–	On CT data	–
Matrix (pixels)	192×192	512×512	169×169	512×512
Resolution (mm)	3.65×3.65	1.37×1.37	4×4	1.37×1.37
Slice thickness (mm)	3.27	3.75	4	4
Slices	–	64	–	64

3 Radiomics discrepancies

Table S 7. Descriptive Statistics and statistical comparisons of radiomics variables in terms of mean values and standard deviations for the two cohorts.

	**Institution 1**		**Institution 2**		
Variables	**Mean**	**Std_Dev**	**Mean**	**Std_Dev**	**p-value**
CONVENTIONAL_SUVmin	3.7737	1.8616	3.3597	1.6822	<<**0.01**
CONVENTIONAL_SUVmean	6.1546	2.8542	5.5975	2.4896	<<**0.01**
CONVENTIONAL_SUVstd	1.4715	0.7308	1.3788	0.6578	**0.0054**
CONVENTIONAL_SUVmax	10.2264	4.7974	9.4158	4.2288	<<**0.01**
CONVENTIONAL_SUVpeak	2.3524	4.2680	5.9848	3.9711	<<**0.01**
CONVENTIONAL_TLG	82.1013	230.5903	75.5440	149.7817	<<**0.01**
HISTO_Skewness	0.5872	0.4147	0.6171	0.4045	0.1229
HISTO_Kurtosis	2.8807	0.8532	2.8957	0.8498	0.2132
HISTO_ExcessKurtosis	-0.1192	0.8532	-0.1042	0.8498	0.2132
HISTO_Entropy_log10	1.1423	0.2083	1.1269	0.1763	**0.0067**
HISTO_Entropy_log2	3.7946	0.6922	3.7434	0.5858	**0.0067**
HISTO_Energy	0.0944	0.0538	0.0942	0.0423	**0.0073**
SHAPE_Volume	11.6197	24.3370	12.6720	24.6858	<<**0.01**
GLCM_Homogeneity	0.3335	0.1058	0.3327	0.0814	**0.0873**
GLCM_Energy	0.0191	0.0187	0.0167	0.0135	0.293
GLCM_Contrast	40.3799	42.1976	32.4835	29.6706	**0.0437**
GLCM_Correlation	0.3036	0.1836	0.3069	0.1315	0.9423
GLCM_Entropy_log10	1.9241	0.3304	1.9367	0.2623	0.2163
GLCM_Entropy_log2	6.3918	1.0975	6.4336	0.8715	0.2163
GLCM_Dissimilarity	4.4523	2.3504	4.1395	1.8138	**0.0625**
GLRLM_SRE	0.9483	0.0362	0.9505	0.0257	**0.0204**
GLRLM_LRE	1.2597	0.2396	1.2304	0.1672	0.3075
GLRLM_LGRE	0.0065	0.0132	0.0056	0.0059	<<**0.01**
GLRLM_HGRE	516.1915	456.6894	419.2514	402.2381	<<**0.01**
GLRLM_SRLGE	0.0059	0.0107	0.0052	0.0051	<<**0.01**
GLRLM_SRHGE	496.7612	442.7954	400.0534	375.1861	<<**0.01**
GLRLM_LRLGE	0.0102	0.0313	0.0075	0.0117	<<**0.01**
GLRLM_LRHGE	607.9699	539.4094	518.3472	689.0347	<<**0.01**
GLRLM_GLNU	22.8289	42.6938	19.5446	45.4589	0.5206
GLRLM_RLNU	245.5027	441.3303	194.1253	307.1768	**0.0858**
GLRLM_RP	0.9331	0.0454	0.9357	0.0337	**0.0397**
NGLDM_Coarseness	0.0365	0.0224	0.0368	0.0191	0.1317
NGLDM_Contrast	0.3887	0.3496	0.3412	0.2579	0.1648
NGLDM_Busyness	0.2764	0.5719	0.2360	0.4246	0.1134
GLZLM_SZE	0.6168	0.1427	0.6097	0.1149	**0.0236**
GLZLM_LZE	102.2394	772.5381	88.0518	1134.3479	**0.0463**
GLZLM_LGZE	0.0066	0.0133	0.0057	0.0057	<<**0.01**
GLZLM_HGZE	510.9761	437.2528	411.2058	377.2074	<<**0.01**
GLZLM_SZLGE	0.0033	0.0053	0.0032	0.0025	<<**0.01**
GLZLM_SZHGE	353.6975	349.1995	276.7329	307.0125	<<**0.01**
GLZLM_LZLGE	2.7491	30.4856	1.5488	19.6571	**0.0014**
GLZLM_LZHGE	12582.0082	42514.1309	12686.7792	93404.5481	**0.0011**
GLZLM_GLNU	7.4366	9.9953	6.4751	6.4384	0.6327
GLZLM_ZLNU	49.2334	87.4316	35.8290	47.5280	**0.0509**
GLZLM_ZP	0.4574	0.1997	0.4445	0.1594	**0.0287**

4 Results of experiment 3

Table S 8. Experiment 3 results: performance of the Cox proportional hazard models trained and tested in cross-validation using different patient representations. Each modality – i.e. radiomics, Dual AD-AE embeddings, ComBat-based standardization, ReComBat-based standardization, and OPNested-based standardization – is fed in the survival model according to three different patient representations: (1) the patient is described by the centroid of its point cloud ("centroid representation"), (2) the patient is described by the topological characteristics of its point cloud ("cloud description representation") and (3) the patient is described by both the centroid and the topological characteristics of its point cloud ("complete representation"). Best values are highlighted in bold.

Models	Centroid representation		Cloud description representation		Complete representation	
	C-index (train)	C-index (test)	C-index (train)	C-index (test)	C-index (train)	C-index (test)
**Radiomics**	0.6962 $\pm$ 0.0190	0.4344 $\pm$ 0.1779	0.5828 $\pm$ 0.0141	0.5184 $\pm$ 0.1203	0.7077 $\pm$ 0.0207	0.4026 $\pm$ 0.1460
**Dual AD-AE**	**0.7803 $\pm$ 0.0132**	**0.6100 $\pm$ 0.1382**	**0.6728 $\pm$ 0.0135**	**0.6481 $\pm$ 0.1305**	**0.7803 $\pm$ 0.0132**	**0.6100 $\pm$ 0.1382**
**ComBat-center-scanner**	0.7041 $\pm$ 0.0201	0.5525 $\pm$ 0.1358	0.5611 $\pm$ 0.0162	0.5101 $\pm$ 0.1228	0.7173 $\pm$ 0.0180	0.5063 $\pm$ 0.1279
**ComBat-scanner-center**	0.7016 $\pm$ 0.0195	0.5366 $\pm$ 0.1266	0.5625 $\pm$ 0.0159	0.5075 $\pm$ 0.1262	0.7291 $\pm$ 0.0185	0.5066 $\pm$ 0.1234
**ReComBat-center-scanner**	0.7064 $\pm$ 0.021	0.5595 $\pm$ 0.1344	0.5621 $\pm$ 0.0161	0.5128 $\pm$ 0.1239	0.7153 $\pm$ 0.0186	0.5201 $\pm$ 0.1224
**ReComBat-scanner-center**	0.7019 $\pm$ 0.0194	0.5444 $\pm$ 0.1240	0.5635 $\pm$ 0.0160	0.5101 $\pm$ 0.1244	0.7260 $\pm$ 0.0192	0.5120 $\pm$ 0.1348
**OPNested ComBat**	0.7019 $\pm$ 0.0194	0.5444 $\pm$ 0.1240	0.5635 $\pm$ 0.0160	0.5101 $\pm$ 0.1244	0.7260 $\pm$ 0.0192	0.5120 $\pm$ 0.1348

5 Software

At this link, the code for Dual AD-AE is distributed. We implemented the training of the dual AD-AE model, adhering to conventional practices involving several procedural steps. Following data preparation and model architecture definition, the training loop iterated through the training data. In each iteration, a batch of input data underwent encoding to derive latent representations, which were subsequently passed through the decoder to generate reconstructed data. The loss function was employed to quantify the dissimilarity between the input and reconstructed data, and backpropagation facilitated the computation of gradients pertaining to the model parameters. These gradients, in turn, drove the updating of model weights through the chosen optimizer. Periodic utilization of the validation set allowed for performance evaluation and the monitoring of training progression, facilitating early stopping to counteract overfitting.

The choice to employ a unique validation set with multiple epochs, rather than opt for cross-validation involving numerous train/test splits with fewer epochs, was underpinned by various considerations. These encompassed the size of our dataset, the inherent nature of the problem, and available computational resources. Although this approach harbored the potential for overfitting and a potentially less precise evaluation of model generalization, its implementation was both straightforward and computationally efficient, given its reliance on the complete dataset. It further provided a singular model framework endowed with a consistent validation set, thereby enabling continuous monitoring of performance dynamics.

However, a cross-validation approach can be interesting as an other training option, particularly maximizing the utility of limited data and furnishing a more robust assessment of generalization, thus we subsequently introduced an alternative training regimen for the AD-AE model. In this regimen, we implemented a cross-validation setup comprising 50 splits, each spanning 100 epochs.
